# Protein Domain Analysis of *C. botulinum* Type A Neurotoxin and Its Relationship with Other Botulinum Serotypes

**DOI:** 10.3390/toxins2010001

**Published:** 2009-12-24

**Authors:** Shashi K. Sharma, Uma Basavanna, Hem D. Shukla

**Affiliations:** 1 Center for Food Safety and Applied Nutrition, Food and Drug Administration, College Park, MD 20740, USA; Email: uma.basavanna@fda.hhs.gov; 2 Department of Biology, Johns Hopkins University, 3400 North Charles Street Baltimore, MD 21218, USA; Email: hshukla2@jhu.edu

**Keywords:** protein domain, neurotoxin, BoNT serotypes, coiled-coil domain, phylogenetic

## Abstract

Botulinum neurotoxins (BoNTs) are highly potent poisons produced by seven serotypes of *Clostridium botulinum*. The mechanism of neurotoxin action is a multistep process which leads to the cleavage of one of three different SNARE proteins essential for synaptic vesicle fusion and transmission of the nerve signals to muscles: synaptobrevin, syntaxin, or SNAP-25. In order to understand the precise mechanism of neurotoxin in a host, the domain structure of the neurotoxin was analyzed among different serotypes of *C. botulinum*. The results indicate that neurotoxins type A, C, D, E and F contain a coiled-coil domain while types B and type G neurotoxin do not. Interestingly, phylogenetic analysis based on neurotoxin sequences has further confirmed that serotypes B and G are closely related. These results suggest that neurotoxin has multi-domain structure, and coiled-coil domain plays an important role in oligomerisation of the neurotoxin. Domain analysis may help to identify effective antibodies to treat Botulinum toxin intoxication.

## 1. Introduction

The seven Botulinum neurotoxins (BoNT/A-/G) are bacterial metalloproteases that act in the cytosol of cholinergic nerve terminals. They cleave core proteins of the neuroexocytosis apparatus causing a long-lasting inhibition of neurotransmitter release [1].BoNTs are extremely toxic proteins of 150 kDa, and consist of a 100 kDa heavy chain and a 50 kDa light chain linked through a disulfide bond [2]. The potency of BoNT/A was determined to be 0.4 × 10^8^ i.p. LD_50_ per mg [1].The discovery that BoNTs are Zn^2+^-endopeptidases [3] has led to identification of several target proteins, which are critical for the docking and fusion of synaptic vesicles to the plasma membrane in the neurotransmitter release process.VAMP (Vesicle Associated Membrane Protein), SNAP-25 (synaptosome associated protein of 25 kDa) and syntaxin form the SNARE complex during docking of synaptic vesicles to the plasma membrane. Different BoNTs proteolytically cleave synaptic proteins. For example, BoNT/B, BoNT/G, BoNT/D and BoNT/F cleave VAMP, BoNT/A, BoNT/C and BoNT/E, cleaves SNAP-25 and BoNT/C cleave syntaxin as part of their mode of action to block neurotransmitter release [4].

Protein-protein interactions play a central role in many cellular functions, and as whole-genome data accumulates, computational methods for predicting these interactions become increasingly important. Computational methods have proven to be a useful first step for rapid genome-wide identification of putative protein structure and function. In the genomic era, one of the most interesting and important challenges is to understand protein interactions on a large-scale. The physical interactions between protein domains are fundamental to the workings of a cell: in multi-domain polypeptide chains, in multi-subunit proteins and in transient complexes between proteins that also exist independently. Protein domain interactions are essential to the functioning of individual cells and whole organisms by acting in several ways: domain-domain interactions in multi-domain polypeptide chains, inter-chain protein interactions in obligate complexes such as multimers or oligomers and in transient complexes between proteins that can also exist independently. Therefore, it is not surprising that protein interactions have been extensively investigated using various methods. 

The parallel two-stranded α-helical coiled-coil is the most frequently encountered subunit oligomerization motif in proteins. The simplicity and regularity of this motif have made it an attractive system to explore some of the fundamentals of protein folding and stability and in testing the principles of *de novo* design [5]. Despite its simplicity, it is a highly versatile folding motif; coiled-coil-containing proteins exhibit a broad range of different functions related to the specific 'design' of their coiled-coil domains. The architecture of a particular coiled-coil domain determines its oligomerization state, rigidity and ability to function as a molecular recognition system [6]. 

In this study, we have examined the protein domains of seven serotypes derived from published amino acid sequence of BoNTs serotypes. All BoNT serotypes have common protein domains present except BoNT/B and BoNT/G. Our analysis provides evidence that BoNT/B and BoNT/G do not have coiled-coil domains and they appeared to be derived from a common ancestor

## 2. Results and Discussion

### Multiple sequence alignment, domain analysis and phylogenetic analysis

**Figure 1:**
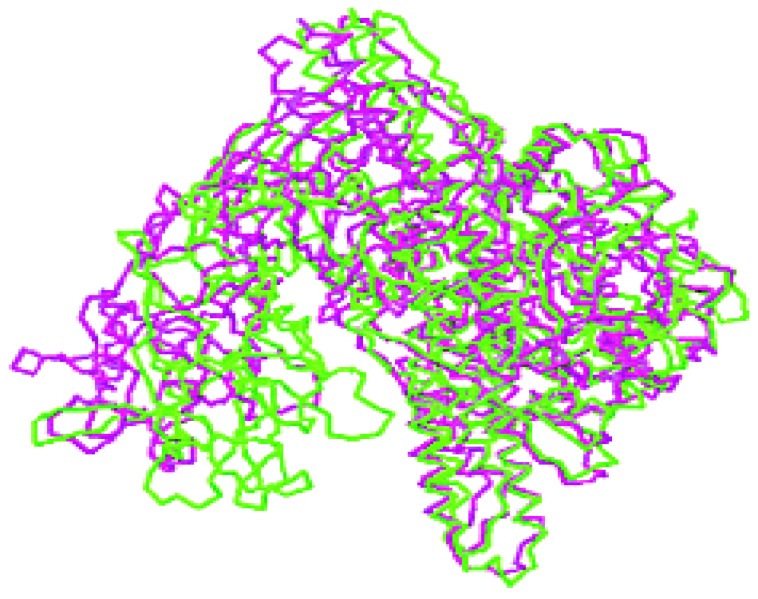
Comparison of BoNT/A and BoNT/B [8] Stereo view of the C-trace of BoNT/A (magenta) superimposed on that of BoNT/B (green). Significant differences in the orientations of the C-terminal halves of each binding domain with respect to the translocation domain are clearly visible (adopted with permission from Swaminathan *et al*. [8]).

The crystallographic structure of BoNT/A and BoNT/B molecules consists of three structural domains matching to three functional domains, namely catalytic, translocation and binding [7]. These three domains are arranged in a linear fashion with the translocation domain in the middle. An overlay of BoNT/B holotoxin with BoNT/A holotoxin indicates that the translocation domain belt has a different position in serotype B. The altered position of the belt in BoNT/B increases exposure of the active site relative to BoNT/A holotoxin [8]. The structure of the binding domain is very similar to that of the C-fragment of tetanus toxin and the binding domain of BoNT/A [9,10]. In BoNT/B, the translocation domain consists of two long helical regions, each 105 Å long, that form coiled coils. However, this region does not adopt a helical conformation. The only α-helix in this region is from residues 638-645 which is in the middle part of the molecule. BoNT/A translocation domain differs from BoNT/B, especially at residues 481-532 that wrap around the catalytic domain, as to keep the catalytic domain in position and is known as belt region. The crystallographic data indicate that only 49% of the residues match in BoNT/A and BoNT/B, indicating that the association of the three domains may be different in the two molecules [8], (Figure 1). There is even a lower sequence similarity (24%) of the belt region among the seven serotypes and the related clostridial toxins [11,12].

The coiled-coil structure is used by nature to stabilize α-helices in proteins. This is possible through a very efficient burial of hydrophobic side chains in proteins so that for the most part, they are not accessed by polar water molecules. Because of this structure, the helices are quite stable. Many structural proteins both inside and outside of cells (keratins, tropomyosin, laminin) have a coiled-coil domain. There seem to be basic, but essential, features that are common to coiled-coil peptides. First, the overall secondary structure is alpha helical and secondly, the hydrophobic residues are arranged on one side of the helices. The typical positioning of the hydrophobic residues in coiled-coils, (*i.e.*, the coiled-coil motif), can be often recognized from the primary structure of the protein. One coiled-coil motif (4-3 hydrophobic repeats) is a heptads repeat of amino acids from A to G so that A and D are hydrophobic and E and G predominantly polar. 

**Table 1:** Coiled coil region in the sequence of botulinum neurotoxins. The amino acid number indicates start and end of the residues.

***BoNTs***	***Position of Coiled coil domain in sequence***
BoNT/A	717-744
BoNT/B	Absent
BoNT/C	753-773
BoNT/D	750-770
BoNT/E	697-717
BoNT/F	716-736
BoNT/G	Absent

The existence of a coiled-coil structure in close proximity to the translocation domain of BoNT/A, however, raises important questions concerning its exact function for blocking the synaptic transmission. Except for BoNT/B and BoNT/G, all other serotypes contain coiled-coil regions within the *N*-terminal region of the heavy chain. The *C*-terminal part of the heavy chain (HC) binds to the surface of target nerve cells and the *N*-terminal part (HN) is involved in the translocation the light chain across the membrane [13]. The two sections of heptads repeats (718 to 750 and 755 to 792) in BoNT/A have been reported previously [14]. These two coiled-coil regions are predicted to be involved in membrane translocation. The existence of oligomeric formation has been reported in BoNT/A, E and Tetanus toxin (TeNT) based on native gel electrophoresis and chemical cross-linking [15,16]. CD analysis of BoNT/A heavy chain indicates that it undergoes an oligomeric transition with the lowering of pH [17].

**Figure 2:**
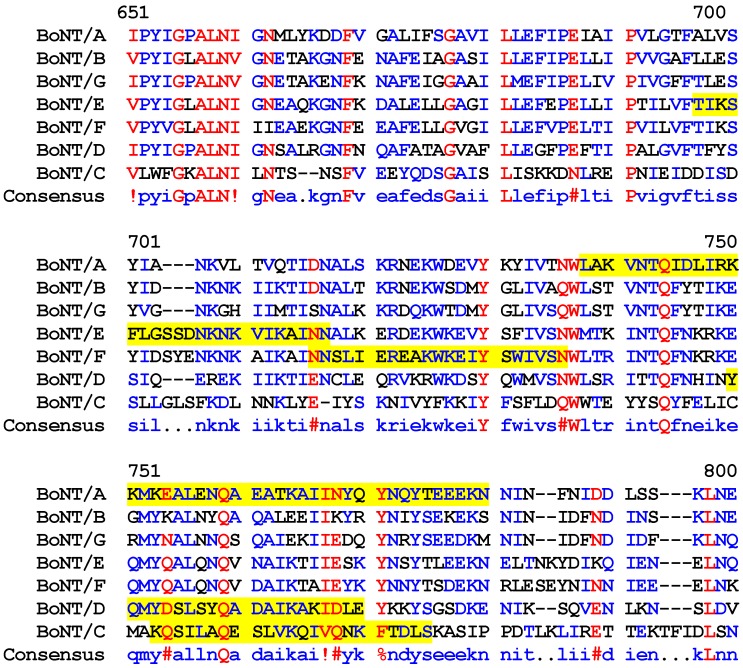
Alignment of BoNT/A protein sequence with the known protein sequences of other serotypes. The yellow highlighted residues are coiled-coil regions

It has been proposed that the belt region, a 54 amino acid segment at the *N*-terminus of heavy chain that wraps around the catalytic domain might act as a chaperone assisting in light chain unfolding, channel transit, and refolding [18,19]. BoNT/C also aggregates into dimer or oligomer during channel formation [20,21]. The SNAP-25 adopts partial secondary structure upon binding to the light chain in the binary complex crystal structure and occupies a similar position as the belt in the holotoxin crystal structure of both BoNT/A and BoNT/B [18]. This hypothesis was considered based on the crystal structure of BoNT/A and BoNT/B belt region, and structure of SNAP-25 bound to BoNT/A L-chain. This observation contradicts our data on the prediction of absent coiled coil domain in BoNT/B, and thus its potential role in differential mechanism of translocation in BoNT/A and BoNT/B. BoNT/B has been reported to form a dimer [20]. However, the *N*-terminal part which is involved in the translocation,in BoNT/A differs from BoNT/B, especially at residues 481-532 that wrap around the catalytic domain, we think that these differences should be taken into account inferring any common translocation mechanism of all BoNTs. However, absence of coiled-coil in BoNT/B and BoNT/G (Table 1, Figure 2) indicates that either oligomerization may not be involved in the channel formation or a different mechanism of channel formation operates in these serotypes. 

**Figure 3:**
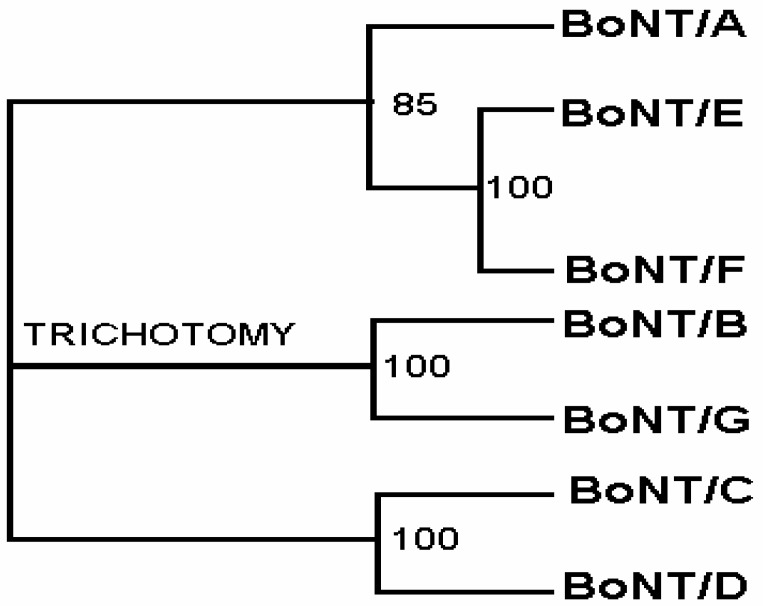
Neighbor joining (NJ) tree for 7 sequences BoNT/A to BoNT/G. Numbers correspond to percentage of times that a particular topology appeared at a particular node in 100 bootstraps. Phylogenetic analysis was performed using CLUSTAL X 1.81 for multiple sequence alignment.

On the contrary, the presence of coiled-coils in a majority of serotypes indicates that oligomerization may be playing a functional role beyond translocation within the cell. The neighbor joining tree for seven serotypes also indicates that BoNT/B and BoNT/G derived from common ancestor (Figure 3).

Protein receptors for BoNT/B and BoNT/G, (synaptotagmins I and II), bind to a pocket at the tip of the *C*-terminal domain of the *C*-terminal fragment of the heavy chain. This corresponds to the unique second carbohydrate binding site of tetanus neurotoxin, the sialic acid binding site [22,23]. In addition, BoNT/B and BoNT/G appears to exhibit lower but same affinity to both synaptotagmins *in vitro*. Similarity calculation based on the *C*-terminal domain of the *C*-terminal fragment of the heavy chain domain amino acid alignment resulted in 43.8% identity scores between them and every other BoNTs [20]. 

While there appears to be a similarity in the receptor binding property of BoNT/B and BoNT/G which is mediated by *C*-terminal domain of the *C*-terminal fragment of the heavy chain, we believe that *N*-terminal part which involve the translocation of the light chain across the membrane could be playing a different role in these two serotypes. Note that our computational predictions lack biochemical evidences and do not reach yes-or-no decisions based on a threshold value. Rather, it yields a set of probabilities that presumably reflect the coiled-coil forming potential in BoNTs published sequences. This means that even at high probabilities there will be (and should be) sequences that in fact do not form a coiled coil, though they may have the potential to do so in a different context. Moreover, the program we used to predict coiled coils cannot predict on buried structures. 

Under physiological conditions, the L-chain is believed to separate from the H-chain at the endosomal membrane, and the L-chain is only active after reduction of the disulfide bond. To release the L-chain, we believe that it is possible that self oligomerization of the H-chain (probably the *N*-terminal part) causes conformational changes there by releasing the L-chain after a disulfide reduction. 

## 3. Experimental Section

### 3.1. Multiple sequence alignment and domain analysis

The sequence analysis of BoNTs was performed in two steps: (I) protein sequences from seven serotypes were obtained from NCBI and analyzed for sequence comparison using the GCG program (Accelrys GCG, Formally known as the GCG Wisconsin Package). (ii) multiple sequence alignment was performed using CLUSTAL V based on algorithm of Higgins [24]. The domain analysis was performed on a Pfam-based PRODOM database using multiple sequence alignments. The Hidden Markov Model was used for domain analysis [25,26].

### 3.2. Phylogenetic analysis

Phylogenetic analysis of seven serotypes of *C. botulinum* neurotoxins was performed using CLUSTALX 1.81 for multiple sequence alignment and Tree-View for neighbor joining tree construction with maximum bootstrapping option [27]. A majority rule consensus tree was generated. Values of greater than 80 to 85% were considered strong support and were considered equivalent to high-confidence values obtained by bootstrap analysis.

## 4. Conclusions

These results indicate that neurotoxin has multi-domain structure, and coiled-coil domain plays an important role in oligomarization of the neurotoxin. Domain analysis may help to identify effective antibodies to treat Botulinum toxin intoxication. A detailed understanding of the architecture, catalysis, and recognition properties of these domains will also help to reveal how the toxins achieve functional diversity.
